# Animal welfare risks from commercial practices involving cephalopod molluscs and decapod crustaceans

**DOI:** 10.1017/awf.2025.25

**Published:** 2025-04-22

**Authors:** Heather Browning, Charlotte Burn, Alexandra K Schnell, Andrew Crump, Jonathan Birch

**Affiliations:** 1 Department of Philosophy, University of Southampton, UK; 2 Centre for Philosophy of Natural and Social Science, London School of Economics and Political Science, UK; 3 Department of Pathobiology and Population Sciences, Royal Veterinary College, UK; 4 Department of Psychology, University of Cambridge, UK

**Keywords:** animal welfare, aquaculture, cephalopod mollusc, decapod crustacean, sentience, slaughter, stunning

## Abstract

There is increasing recognition that the welfare needs of cephalopod molluscs and decapod crustaceans are important. Current commercial practices involving these animals include a range of potential threats to their welfare, such as conditions of farming, capture, transport, and slaughter. This article draws from and updates our 2021 review for the UK Government, recommending a range of relatively simple and impactful changes that could benefit welfare while highlighting important research gaps that should be prioritised to facilitate the drafting of guidelines for best-practice.

## Introduction

There is increasing recognition that the welfare needs of cephalopod molluscs (e.g. octopus, squid, cuttlefish) and decapod crustaceans (e.g. lobsters, crabs, shrimp) are important. In the UK, these groups of invertebrate animals were recently recognised as sentient as part of the Animal Welfare (Sentience) Act 2022. We here take sentience as the capacity for subjective experience, the ability to experience a range of feelings or ‘affects’, such as pain, joy, comfort, hunger, and contentment (Browning & Birch 2022). The inclusion of cephalopod molluscs (henceforth, ‘cephalopods’) and decapod crustaceans (henceforth, ‘decapods’) followed a thorough review of the current evidence (over 300 scientific studies) assessed against a set of physiological, cognitive, and behavioural criteria specifically relating to the capacity for pain experience (Birch *et al.* 2021; Crump *et al.* 2022).

This is not to say that the evidential picture is definitive: it is complex, incomplete and requires a range of inferences and background assumptions. There is ongoing disagreement regarding how to interpret the evidence and whether to consider cephalopods and decapods as sentient (e.g. Mason & Lavery 2022; see also the range of responses to Crump *et al.* 2022). Nevertheless, it has been taken by the UK Parliament to justify legislative protection of these animals. Elsewhere, cephalopods have been recognised in animal welfare law, including the UK’s Animals (Scientific Procedures) Act 1986, New Zealand’s Animal Welfare Act 1999, and similar legislation in the EU, Canada and some Australian states. Decapods have more limited recognition but still receive certain protections in New Zealand, Austria, Norway, Switzerland, and some Australian states. Recognising these animals as sentient requires, at minimum, that we ask: which current practices could potentially harm their welfare?

In this article we survey the potential welfare harms arising from current commercial practices involving cephalopods and decapods used mainly for food. Throughout the article, where we refer to potential suffering or other welfare harms, this is taken to be conditional on the sentience of the animals in question. As we have highlighted above we take there to be strong evidence in favour of sentience in both cephalopods and decapods, but this is still open to a degree of uncertainty. The article draws on, updates and expands our review for the UK Government (Birch *et al.* 2021). Where possible, we have relied on literature that directly studies the welfare impacts of different practices, however as such literature is limited, we have also inferred welfare harms from relevant changes in behaviour, health, and physiology (e.g. those indicative of stress responses). Our aim is not to draw up best-practice guidance for the treatment of cephalopods and decapods, but to highlight some areas where guidance, research and/or policy interventions may be required. While we include recommendations for easy, near-term changes, our primary aim is to raise awareness of key issues and encourage further discussion regarding the best ways to improve welfare in these and other areas. The following two sections survey the range of potential welfare harms to cephalopods and decapods, respectively. *Recommendations and evidence gaps* highlights what we see as the easiest and most beneficial changes to start with, as well as where more research would be most valuable. The final section concludes the discussion and highlights the key animal welfare implications.

## Commercial practices involving cephalopods

### Cephalopods in sea fisheries

Most commercially used cephalopods are wild-caught and slaughtered. Wild capture involves a range of potential welfare harms, most notably the capture methods themselves, inappropriate housing after capture, and inhumane methods of slaughter. Wild-caught cephalopods, if not dead upon landing, usually die after being taken from the water, posing significant welfare risks due to physical trauma and asphyxiation between point of capture and landing, a journey that can take hours or even days. The welfare issues are similar to those arising for wild-caught fish. There is no easy way to mitigate these risks, but codes of best practice should be developed for cephalopods caught and landed alive (for recommendations, see Pieroni *et al.* 2022; Sykes *et al.* 2024).

Globally, many inshore cephalopod fisheries target octopus, cuttlefish, and squid species (Pierce *et al.* 2010). Capture methods vary across fisheries and include netting, trapping, and dredging techniques. Unlike decapod fisheries, captured cephalopods are not transported alive and thus welfare risks for live maintenance and captivity are not considered here. This section will instead focus on welfare risks *from the point-of-capture to landing.* There is little scientific literature explicitly identifying the welfare implications of cephalopod fisheries. Consequently, the welfare risks discussed in this section are largely based on capture, handling, and transport data from studies that have captured cephalopods for scientific purposes.

Squid are caught using trawls, driftnets, and seine nets. Hand-jigging is also commonly used in squid fisheries (Pierce *et al.* 2010). Squid caught in nets are typically dead when brought abroad, whereas squid caught through jigs are alive. Hand-jigging is considered one of the more humane methods for live capture because it usually causes less harm to the squid, though it may not be suitable for all species and can cause damage to skin or loss of arms and/or tentacles through autotomy or improper handling when bringing aboard (Cabanellas-Reboredo *et al.* 2011). While other live capture methods exist, jigging is often preferred for minimising physical injury (Pierce *et al.* 2010). Jigging is also selective in the size range of animals captured (Rathjen 1991), reducing the need to discard undersized animals. However, post-landing methods of slaughter are not always considered humane, raising ethical concerns about the overall treatment of squid during and after capture.

Octopus and cuttlefish are primarily caught using trawls, pots, and traps (Pierce *et al.* 2010). Cuttlefish can also be caught using nets (i.e. gillnets and trammelnets) and octopus can be caught as by-catch in pots and traps. Trawled or netted cephalopods are usually brought aboard the vessel dead or nearing death, whereas trapped animals are caught alive (industry sources). Dredging has also been used as a capture method for octopus, cuttlefish, and squid. Trawling and dredging are the most environmentally destructive methods and, in some instances, undersized cephalopods are discarded, already dead (Pierce *et al.* 2010).

The following subsections will discuss the potential welfare risks associated with the different capture methods.

#### Physical trauma

Capture techniques can result in physical trauma to cephalopods. Specifically, physical trauma might arise from rough handling, causing the mantle to detach from the head (AK Schnell, personal observation 2013). Raising benthic species from depth too quickly can lead to buoyancy malfunction due to rapid changes in pressure (Forsythe *et al.* 1991; Sherrill *et al.* 2000; McDonald 2011). However, unlike the swim bladder of fish, the buoyancy mechanism in cuttlefish (the cuttlebone) is unpressurised, so its volume is not markedly altered as the animal changes depth (Denton & Taylor 1964; Sherrard 2000). Nevertheless, rapid vertical movement may cause air to be trapped inside the mantle cavity (AK Schnell, personal observation 2013) resulting in potential discomfort or pain.

During capture methods that involve nets, individuals might be pursued to exhaustion and then suffocate and become crushed under the weight of other animals. However, further research is required to determine the severity of this risk. Finally, collision with other animals or the side of the net routinely causes skin damage (Boyle 2010). Cephalopods have soft skin and are particularly susceptible to skin ulcerations and fin injuries (i.e. specific to cuttlefishes and squids as octopuses do not have fins) that can result in permanent damage. These injuries encourage bacterial growth (Gestal *et al.* 2019) and can lead to disease or death (Hanlon *et al.* 1984; Boyle 2010; Gestal *et al.* 2019).

Although most netted animals will die during or quickly after being hauled up, skin and fin injuries become a welfare concern if: (i) live individuals are left in nets for hours or days prior to landing (as can be the case with trawl and drift nets); if (ii) live undersized animals are released back into the water with injuries; and/or (iii) if the skin injuries cause the animals to experience pain prior to death. Skin plays a vital role in the survival of cephalopods as they use body patterns for both concealment and communication (Hanlon & Messenger 2018). Moreover, minor injuries in squid increases the risk of predation (Crook *et al.* 2014), and squid with skin and fin injuries respond poorly to temperature and salinity changes compared to uninjured squid (Hanlon *et al.* 1983). Using soft netting material or alternative capture methods (i.e. traps or jigging) might decrease some risk of physical trauma involved in netting capture methods (Iglesias *et al.* 2007), but this has not been systematically tested.

#### Aggression and cannibalism

Except for a few species, both octopods and cuttlefish are relatively solitary animals. Confinement in a small space with conspecifics, such as a pot or trap, might not only cause stress but also fighting. Indeed, limb amputation is commonly observed in wild-caught octopuses (Florini *et al.* 2011), which might be a result of either autophagy/auto-mutilation (Reimschuessel & Stoskopf 1990; Budelmann 1998) or fighting. Another risk is cannibalism; all coleoid cephalopod groups have cannibalistic tendencies, particularly between individuals that are not size-matched and when insufficient food is provided (Aguado-Gimémenz & Garcia Garcia 2002; Hayter 2005; Moltschaniwskyj *et al.* 2007; Budelmann 2010; Ibáñez & Keyl 2010; Pierce *et al.* 2010; Jacquet *et al.* 2019).

Consequently, fisheries that include traps or pots to detain live individuals together should ensure that their devices are large enough for the species in question, baited with sufficient prey to sustain the maximum number of captive individuals and be checked frequently. Leaving the devices *in situ* for several days can result in discomfort, stress, and even death, as the confined space can provoke trapped animals to fight or eat each other. The frequency of checking octopus traps varies depending on local regulations, fishing practices, and environmental conditions. In False Bay, South Africa, for example, soak time, trap density, and sea surface temperature are considered when determining optimal intervals (Sanjay 2022). Additionally, variations in octopus behaviour, such as differences in trap interaction and escape attempts, suggest that checking frequency may need to be adjusted to account for activity patterns and catch efficiency (Dominguez-Lopez *et al.* 2021).

#### Exposure to inappropriate salinity and temperatures

Cephalopods are highly stenohaline and stenothermal (Fiorito *et al.* 2015), meaning they can only tolerate a narrow range of salinity and temperature (Moltschaniwskyj *et al.* 2007). Changes in salinity can result in visual indicators of stress or discomfort, such as blanching of the skin and excessive inking, and can lead to death (AK Schnell, personal observation 2012). While stratification or mixing can sometimes buffer the effects of freshwater run-off, cephalopods in shallow or enclosed inshore environments remain vulnerable to sudden salinity changes. A single overnight rainfall event of over 60 mm caused salinity fluctuations severe enough to result in up to 70% mortality in cuttlefish held in open-air, pot-like aquarium traps exposed to the elements (AK Schnell, personal observation 2012). Such conditions, likely to become more frequent with extreme weather, highlight the need for frequent trap checks to minimise mortality.

#### Slaughter methods

Trawled or netted animals are usually brought aboard dead, whereas trapped or jigged animals are often landed alive (industry sources). If the animal is still alive, it will die from asphyxiation before being iced. Time of death via asphyxiation on boats is not well-documented but can take minutes to hours, which raises welfare concerns, especially since cephalopods are likely to experience distress during the process. Anecdotal evidence suggests that potentially inhumane methods are sometimes used on fishing vessels, such as clubbing, slicing the brain, and reversing the mantle (Pereira & Lourenco 2014).

There is currently an evidence gap regarding humane slaughter methods that are commercially practical and available. There are efforts to improve and standardise euthanasia for captive cephalopods used in scientific experiments (Andrews *et al.* 2013; Fiorito *et al.* 2015; Butler-Struben *et al.* 2018). Currently, the only recommended method of humane slaughter for cephalopods is terminal overdose of an anaesthetic (typically magnesium chloride and ethyl alcohol), often followed by decerebration (Boyle 2010; Andrews *et al.* 2013; Fiorito *et al.* 2015; Abbo *et al* 2021). However, this would be inappropriate for cephalopods slaughtered for human consumption. Furthermore, mechanical methods that do not involve contamination, such as cutting or puncturing of the brain, require skilled practitioners to ensure they are performed correctly (Boyle 2010; Andrews *et al.* 2013; Fiorito *et al.* 2015) and are inefficient for large-scale practices. Further research is needed to determine the optimal slaughter methods for commercial cephalopod fisheries. We have been unable to find any codes of best practice or voluntary guidelines specific to cephalopod fisheries. Even though cephalopods are often caught as by-catch, it would be sensible to develop codes of best practice for circumstances in which cephalopods are alive when landed.

### Octopus aquaculture

Currently, cephalopod aquaculture is small-scale and only performed with a few species (O’Brien *et al.* 2018). However, farms can be found in Europe, Australia, Latin America and Asia (Jacquet *et al.* 2019). Cephalopods are sometimes suggested as an attractive candidate for large-scale commercial aquaculture. This is due to increasing demand for cephalopod consumption, their high value, rapid growth, high food conversion rate, protein content, and fecundity (Pierce *et al.* 2010). Both common cuttlefish (*Sepia officinalis*) and common octopus (*Octopus vulgaris*) have been identified as promising candidates for commercial aquaculture in Europe, and some progress has been made in farming *O. vulgaris* in Spain. The issue of octopus welfare in aquaculture has become more pressing with recent proposals to develop larger commercial farms in the Canary Islands, with worries about animal welfare at the forefront, alongside concerns about sustainability (Marshall 2023). Already some states in the US have issued pre-emptive bans on octopus farming and import of farmed octopus. The following sections will outline key welfare risks arising from octopus farming, highlighting the features of octopus that make them especially unsuitable for intensive conditions and the lack of humane slaughter methods.

#### Hatchling mortality

One of the currently limiting issues in captive management of octopus is hatchling mortality. As well as limiting the viability of cephalopod farming, this can also be a welfare issue. For *O. vulgaris*, survival rates are, at best, around 30–40% at day 40 (Iglesias *et al.* 2007) and < 10% by day 60 (Vaz-Pires *et al.* 2004). This is primarily due to problems with temperature, water quality, and nutrition (Vaz-Pires *et al.* 2004; Boyle 2010; Navarro *et al.* 2014). Moreover, hatchlings require a large amount of live food (larval shrimp and other crustacea), which can be difficult to obtain (Iglesias *et al.* 2007; Pierce *et al.* 2010). Young animals dying of poor nutrition and inappropriate housing conditions are highly likely to suffer poor welfare.

#### Capture and transport

As captive breeding efforts and rearing have tended to fail, octopus aquaculture often takes the form of ‘ranching’ or ‘rearing’, in which young animals are captured and grown in captive tanks for eventual sale. As noted above, many capture and transport methods can harm cephalopods. Cephalopods require highly oxygenated water, and prolonged transport can lower oxygen and increase nitrogenous waste. An air stone or aerator should be used whenever possible (Iglesias *et al.* 2007; McDonald 2011; Fiorito *et al.* 2015). Additionally, if the animals ink in the water and it is not subsequently cleaned (or the animal transferred), the ink can coat the gills and cause asphyxiation (Hayter 2005; McDonald 2011). Several species of octopus show stress-related biomarkers after being caught by trawl, such as a compromised immune system, but they typically recover within 24 h (Barragán-Méndez *et al.* 2019). Some species appear more suited than others to these procedures – for example, *O. vulgaris* and *S. officinalis* show some resistance to stress from handling and transport (Vaz- Pires *et al.* 2004; Cooke *et al.* 2019).

A working group through FELASA (Federation of European Laboratory Animal Science Associations) has recently published a set of best-practices for capture and transport of cephalopods (Sykes *et al.* 2024), though this is primarily for research purposes and may not reflect larger-scale commercial operations.

#### Poor nutrition

Poor nutrition is one of the primary obstacles to establishing large-scale octopus aquaculture, as the animals are carnivorous and typically require live prey (Boyle 2010; Pierce *et al.* 2010; Navarro *et al.* 2014). Although work has been done on developing suitable alternatives, none has been successful enough for widespread use (Pierce *et al.* 2010). As it stands, there is insufficient understanding of the metabolism and nutritional needs of cephalopods to formulate complete diets (O’Brien *et al.* 2018). Animals which fail to thrive on food sources provided will experience a range of welfare harms, such as hunger as well as nutritional and metabolic diseases.

#### Inappropriate housing

The quality of the aquatic environment is critical to cephalopod health and welfare. Cephalopods have precise environmental needs, requiring strict monitoring of oxygen, pH, CO_2_, nitrogenous waste, and salinity levels, as well as rapid removal of ink as necessary (Vaz-Pires *et al.* 2004; Hayter 2005; McDonald 2011; Sykes *et al.* 2012; Fiorito *et al.* 2015; Cooke *et al.* 2019). Inadequate water conditions can lead to health issues, infections, respiratory problems, agitation, frequent inking and jetting, and even death (Hanley *et al.* 1998; Hayter 2005; Fiorito *et al.* 2015).

Other environmental factors, such as lighting, temperature, and noise or vibrations, also significantly impact their welfare (Hayter 2005; Fiorito *et al.* 2015). Cephalopods possess unique sensory abilities, such as detecting polarised light, and advanced mechanoreception and chemosensory capabilities, which demand specific environmental conditions (Browning 2019; Cooke *et al.* 2019). Temperature appears particularly important, impacting feeding, growth, and lifespan (Aguado-Giménez & García García 2002; Sherrill *et al.* 2000).

Inadequate shelter in captivity is a major stressor for cephalopods. In their natural habitat, these soft-bodied molluscs use hiding and rapid escape strategies against predators (Cooke & Tonkins 2015). A lack of sufficient or appropriate shelter can cause behaviours associated with fear and stress, such as inactivity and anorexia (Sherrill *et al.* 2000). Moreover, stress in octopus can even result in autophagy: the consumption of their own limbs (Hayter 2005). Providing ample hiding spots, such as shelters or caves, is essential for their welfare (Vaz-Pires *et al.* 2004).

Furthermore, as discussed above, appropriate social grouping is crucial. Most octopus species are solitary and prone to stress when overcrowded. Housing them individually is important, as crowding can trigger aggression, cannibalism (Aguado-Giménez & García García 2002; Hayter 2005; Budelmann 2010; Pierce *et al.* 2010; Jacquet *et al.* 2019) and increased stress, affecting their resting and feeding habits (Cooke *et al.* 2019).

#### Lack of cognitive stimulation

There is also the potential for poor psychological welfare for captive octopus, due to their behavioural and cognitive complexity (Cooke & Tonkins 2015; Jacquet *et al.* 2019). Octopus are even able to recognise and form relationships with caregivers, and the presence of familiar people can impact their welfare (Narshi *et al.* 2022). Jacquet *et al.* (2019) are concerned about lack of cognitive stimulation for farmed octopus. They worry that the “tightly controlled and monotonous environments” typical of farming do not allow for the cognitive stimulation, exploration, and environmental control necessary for psychological welfare. Cephalopods regularly show signs of stress in poor captive environments, such as irregular swimming patterns, lethargy, agitation, and anorexia (McDonald 2011).

#### Disease

Several factors, including stress, suboptimal water quality, and inadequate nutrition, can predispose cephalopods to disease. Stress, in particular, weakens their immune systems, increasing susceptibility to bacterial, viral, and fungal infections (Sherrill *et al.* 2000; McDonald 2011). The cephalopod immune system is not well understood (Sykes & Gestal 2014; O’Brien *et al.* 2018). Whilst viral infections have rarely been reported, bacterial infections commonly occur in skin lesions (as above), and gills (Sykes & Gestal 2014; Fiorito *et al.* 2015). Parasites are common in wild animals and can appear in captive stocks if live prey are used (Sykes & Gestal 2014). Additionally, the current lack of comprehensive knowledge of cephalopod analgesia and anaesthesia poses welfare concerns, particularly when animals are injured or need to undergo medical interventions (Fiorito *et al.* 2015).

#### Slaughter methods

There is, at present, no established method to humanely slaughter farmed cephalopods; a major evidence gap. Proposals for commercial octopus farms in the Canary Islands have suggested slaughter through immersion in ice slurry, but there is no evidence that this is a humane method for octopus. The use of ice slurry for slaughtering fish has faced criticism due to its potential to cause prolonged distress and pain (Marshall 2023). This occurs as the fish’s body temperature gradually decreases. Additionally, the sudden immersion in extreme cold can lead to thermal shock. In some instances, this method also results in asphyxiation, as fish struggle to get sufficient oxygen from the water (Marshall 2023). As noted in the previous section, other more humane methods are not appropriate for use on a commercial scale.

## Commercial practices involving decapods

This section does not aim to provide a comprehensive guide to safeguarding the welfare of decapods in commercial use. Rather, the focus is on identifying specific practices that potentially create a risk of poor welfare, with a focus mainly on marine rather than freshwater decapods, as well as current evidence gaps. In 2022, 62.2% of decapod production consisted of penaeid shrimp species (by live weight equivalent; Food and Agriculture Organisation [FAO] 2024), followed by 23.3% being red swamp crayfish (*Procambarus clarkii*), and 6.4% being Chinese mitten crab (*Eriocheir sinensis*).

### Handling of wild-caught decapods during capture, transport, and sale

Shrimp and prawns are currently farmed in very large numbers (Mood & Brooke 2019), but most commercial use of other marine decapods involves wild-capture and the subsequent transport, sale, and slaughter. Whilst farming comprised 68% of crustacean production in 2022 (FAO 2024) and will be covered below, the following sections first outline the primary welfare risks to wild-caught marine decapods that arise during the processes of transport, sale, and slaughter, including the direct harms from declawing and nicking, harms from inappropriate housing and transport conditions, and poor handling by untrained handlers. An estimated 3.3 million tonnes of shrimps and lobsters were caught from marine capture fisheries worldwide in 2022, which was approximately 9.6% by weight of all marine captured animals (FAO 2024).

#### Accidental injury

It is generally in the interests of the shellfish industry to avoid damaging the decapods they catch, with intact animals fetching a much higher value than injured ones would, especially in larger species. Therefore, industry guidance already emphasises careful handling as good practice (e.g. Jacklin & Combes 2005; Seafish *et al.* 2024). Risk of physical damage is greater for catches that are intended for markets with less emphasis on the quality of individual animals, such as trawl-caught species. Accidental physical injuries to decapods include cracked carapaces, damaged antennae, and loss of limbs. Haemolymph can rapidly leak from cracks, killing the animal. In species intended for relatively prolonged live storage or transport, industry guidance recommends that animals are carefully inspected, and those with damaged limbs should be prompted to cast off the limbs via autotomy (Jacklin & Combes 2005). It is unclear what the relative welfare impact of external injury versus autotomy is to decapods, because injuries vary greatly, but risk of infection or rapid death is lessened with autotomy. Crabs with claws that were manually twisted off showed more defensive behaviour in contests, more frothing at the mouth and more haemolymph loss, compared with those whose claws were autotomised following an incision to the joint above the merus (McCambridge *et al.* 2016).

The risk of accidental injury can be reduced by refined capture methods, such as using smooth plastic inserts in creels to prevent limb tearing when crabs are pulled from netting (Jacklin & Combes 2005), avoiding rapid haulage of lobsters from deeper waters (Basti *et al.* 2010), or where it is not possible to alter commercial haulage speeds or capture depths allowing recovery in recirculating seawater (rather than damp storage) (Basti *et al.* 2010). Studies on species caught using both creels and trawling (e.g. langoustine [*Nephrops norvegicus*] and shrimp [*Pandalus borealis*]) have shown trawling causes greater physiological stress, risk of injury, and mortality, especially when trawl times were longer (Ridgway *et al.* 2006; Albalat *et al.* 2009; Larssen *et al.* 2013). Alongside discussions about the economic and environmental effects of trawling for decapods (e.g. Williams & Carpenter 2016), the welfare risks should also be considered.

There is risk of physical injury (such as crushing) during transport and storage, which can be reduced by using well-designed species-appropriate containers. Containers should be resistant to crushing, not allow limbs to become caught, and not contain so many animals that those above do not crush those below (Barrento *et al.* 2010). When lobsters are stored onboard in totes, they should be packed with their tails curled beneath them to protect their ventral surface from puncture, face in the same direction, and be at a density that aids stability, but without pressing the animals too tightly together (Basti *et al.* 2010).

At all stages, handling decapods causes physiological stress (Jacklin & Coombes 2005) and should be performed with care and kept to a minimum. If decapods are ‘thrown’ (e.g. Barrento *et al.* 2008) or ‘tossed’ (Lavallee *et al.* 2000) into containers, this increases the risk of physical injury and loss of vigour compared with more careful placement. Careless and rough handling is a welfare risk and should be avoided.

#### Declawing

Declawing is the practice of manually twisting or snapping the claws off a decapod, and evidence suggests this may be painful for the animals. Declawing can be contrasted with inducing the animals to autotomise their own claws, usually by making an incision in the limb at certain locations. Declawed crabs will tend their wound, shield it, and in some cases display a ‘shudder’ response (McCambridge *et al.* 2016). They also show a physiological stress response (as indicated by glucose and lactate in the haemolymph) for at least 24 h after the injury, which is more severe for manual declawing than induced autotomy (Patterson *et al.* 2007). Declawed crabs may be thrown back to sea (the claw being the most valuable product in some cases) but without their claws, they are at a competitive disadvantage in contests with other crabs and unlikely to mate (McCambridge *et al.* 2016), as well as less able to gain access to bivalves, one of their main food sources (Patterson *et al.* 2009; Duermit *et al.* 2015). Larger wounds can also lead to death within days (Duermit *et al.* 2015).

It is reasonable to conclude (with high confidence) that declawing true crabs (infraorder Brachyura) causes suffering. UK industry codes of practice discourage declawing (Seafish *et al.* 2024), but declawing is largely unregulated, with bans in place only in some states within the US. The practice was banned in the UK from 1986 until 2000, when the relevant legislation was overridden by a European Union regulation (No 850/98). Banning declawing would be an easy, low-cost intervention to improve the welfare of decapods.

#### Disabling pincers (including nicking)

Decapod pincers or large claws usually require disabling in some way, to prevent injury to both human handlers and other animals sharing the same container. For clawed lobsters, the usual method is to restrain the claws using elastic bands or cable ties (Jacklin & Combes 2005; Seafish *et al.* 2024). Research into the welfare effects of banding is yet to provide any conclusive evidence that this practice compromises lobster welfare compared with the harms of leaving the claws unrestricted. When comparing the haemolymph parameters of group-housed banded American lobsters (*Homarus americanus*) with those of individually housed non-banded lobsters, there was a significant lasting increase in calcium levels but not of glucose and lactate, which are more typically taken to indicate stress (Coppola *et al.* 2019). There is therefore no reason at this stage to consider regulating this procedure.

For brown crabs (*Cancer pagurus*), banding is considered unsuitable within the shellfish industry (Jacklin & Combes 2005; Seafish *et al.* 2024), due to the claws’ tapered conformation. Instead, if live storage or transportation of the crabs is necessary, the tendon connecting the two parts of each claw is cut. This procedure is known as ‘nicking’. There is evidence that this has negative welfare effects. Nicking causes elevated glucose and lactate in the haemolymph and increases the risk of muscle necrosis and pathology (Welsh *et al.* 2013). These effects are worsened at warmer temperatures, whilst colder temperatures helped reduce the risk of physiological stress and pathology (Johnson *et al.* 2016). Due to the health and welfare risks to crabs, alternatives to nicking should be developed and implemented. One potential alternative is immobilisation using elastic bands as is sometimes practised for brown crabs in Norway (Woll *et al.* 2010). In blue crabs (*Callinectes sapidus*), elastic bands can be successfully used for binding claws if a small block or dowel is first gripped between the two dactyls of each claw and then left in place (Haefner 1971). Another solution to prevent fighting could be to use individual compartments for storing crabs, equivalent to the ‘tubes’ used for *Nephrops* (langoustine).

#### Social stress and aggression

Aggression and stress can occur in decapods that are usually solitary in the wild, such as lobsters, when many animals are kept in close proximity, such as being trapped in the same creel or housed in live storage tanks at food retailers. Social aggression can cause ‘anxiety-like’ states in crayfish (*P. clarkii*) (Bacqué-Cazenave *et al.* 2017), and it is reasonable to assume that social aggression will produce similar states in other decapods. While socially grouping lobsters with bound pincers did not significantly increase haemolymph glucose or lactate compared to individual holdings (Coppola *et al.* 2019), this could be due to the protection from injurious aggression or because the stress measures used were not sensitive to the effects. To prevent aggression and associated stress during capture, the Seafish code of good practice for handling crustaceans recommends the use of creels with a second chamber and with escape gaps or a large mesh net (where practical) to allow by-catch to escape (Jacklin & Combes 2005).

Though low stocking density may be important in preventing social stress, one survey conducted in Portugal showed that stocking densities can be very high (maximum reported: 300 kg m^−3^) and sometimes exceed recommendations (120 kg m^−3^; Barrento *et al.* 2008). Carder (2017) investigated live lobster storage conditions at nine UK food retailers and found that lobsters were stocked at densities that caused some individuals to be on top of each other in eleven of the 26 display tanks observed. Indeed, in four tanks, there were at least two full layers of lobsters. Similarly, Crustacean Compassion (2020) reported lobsters fighting in a wholesaler display tank, and up to 50 lobsters being displayed within a single tank (dimensions not given). High stocking densities of socially stored decapods could represent a risk to welfare.

#### Exposure to inappropriate temperatures

The thermal preferences of decapods differ between species and depend to some extent on what temperature they are acclimated to. For most species, their upper and lower temperature tolerance is currently unknown (Lagerspetz & Vainio 2006). However, there are potential welfare risks from exposing animals to temperatures that are too high or low.

Physiological stress, disease susceptibility, and mortality are higher in decapods transported or stored at excessively warm temperatures. This can occur whenever vessel- or shore-based storage containers cannot be cooled to an optimal temperature, such as during warm weather (Lavallee *et al.* 2000; Jacklin & Combes 2005). Studies of decapod transport at different temperatures have shown negative health, behaviour and physiological stress effects at higher temperatures for brown crabs (*C. pagurus*) (Woll *et al.* 2010; Barrento *et al.* 2011; Johnson *et al.* 2016), shrimps (*P. borealis*) (Larsson *et al.* 2013) and Asian tiger prawns (*Penaeus monodon*) (de la Vega *et al.* 2007). Decapods in both immersed and damp storage must, therefore, be kept below a maximum temperature threshold appropriate for their species (Jacklin & Combes 2005). This is the case even for temporary storage (e.g. onboard vessels or awaiting transfer) – for example, lobsters landed on sunny days showed greater loss of vigour than those on cloudy days, presumably because of exposure to sunlight (Lavallee *et al.* 2000). Use of shade covers, running seawater hoses, and planning capture times to avoid the hottest parts of the day can all reduce excessive heat exposure. UK codes of practice recommend storing decapods between 4 and 8^o^C (Seafish *et al.* 2024).

Excessively cold temperatures may also be a problem. Ice or ice-packs are sometimes used to cool decapod environments on board vessels and during live transport, because they reduce the animals’ activity levels and oxygen requirements, helping prolong their lives (Jacklin & Combes 2005). However, ice should not be placed in direct contact with decapods (Seafish *et al.* 2024). Fishing industry reports suggest that the sudden cold can stress and even kill many decapod species from UK waters (Jacklin & Combes 2005). In some countries, including Italy and Switzerland, displaying and transporting live crustaceans on ice or in icy water is illegal. Most marine decapods do not inhabit polar regions (the exception being certain caridean shrimp species), so they would rarely encounter ice in nature, and most become immobile at or below about 2°C (Frederich *et al.* 2002). The reduced activity in decapods when cooled to near freezing is sometimes termed ‘torpor’. It reduces the metabolic rate, which helps them survive short cold periods and regain activity once temperatures increase again. For decapods fished in waters that rarely reach temperatures below 4°C (such as the UK; Morris *et al.* 2018), torpor is unlikely to be a ‘natural’ behaviour.

Whether near freezing temperatures cause nociception or pain in decapods is unknown. Research into this is urgently needed, especially because there is the assumption that extreme cooling has anaesthetic effects, which is in direct conflict with the possibility that it could cause avoidance, nociception, or pain. Even in humans, this paradox exists, because very cold temperatures can cause pain, but can otherwise numb certain other sources of pain (Yin *et al.* 2015), so the situation may also be complex in decapods, and current evidence is inconclusive. Cold shock did not influence haemolymph serotonin or octopamine levels in either crabs or shrimp (Weineck *et al.* 2018). Lobsters (*H. americanus*), spiny lobsters (*Panulirus japonicus*), and prawns (*Penaeus japonicus*) have cold-sensitive neurons in their ventral nerve cord, which increase their firing rate within a temperature range of 0.5–5.5°C (Tani & Kuramoto 1998). Puri and Faulkes (2015) found no evidence for cold-sensitive nociceptors in crayfish (*P. clarkii*), but this study used a much colder stimulus (−78°C) than either conventional chilling methods or ecologically relevant conditions. Cold nociception in general is not well understood across species, and it may have evolved later than heat nociception (Smith & Lewin 2009). This is an important evidence gap: there is a need for better knowledge of the lowest temperature that commercially important species of decapod can tolerate without harming health and welfare.

#### Storage and transport out of water or in marine vivier vehicles

Another potential welfare risk is the open-air storage and transport of aquatic decapods. For instance, rock lobsters (*Panulirus cygnus*) will tail-flip (thought to be a behavioural sign of stress) as soon as they are removed from the water (Paterson & Spanoghe 1997). Some decapods, especially brown crabs, green crabs, and lobsters, can typically survive for 2–3 days in ‘dry’ storage, as long as the conditions are sufficiently damp. This is sometimes known as damp storage or semi-dry storage. Industry guidance suggests that decapods in dry storage should be covered with wet seaweed, fabric, paper or hessian and kept between 4 and 8^o^C (Seafish *et al.* 2024). The welfare effects of dry storage are not well known. One study investigating the effects of damp storage on brown crabs (*C. pagurus*) reported that waste products, such as ammonia, started to accumulate in the haemolymph, since seawater is needed to remove them (Woll *et al.* 2010). This accumulation of waste products may or may not cause suffering — this is an evidence gap.

A key welfare risk to (non-amphibious) decapods is hypoxia (lack of oxygen), which causes lactate to build up in the tissues due to anaerobic respiration. In humans, this build-up of lactate is painful. Whether it is also painful in decapods is unknown — another evidence gap. For animals held in vivier vehicles (lorries, boats or trailers adapted to transport marine life in seawater), industry guidance suggests that oxygen saturation should not fall below 85%, which is a risk over time, at higher stocking densities, and under warmer temperatures (Seafish *et al.* 2024). Hypoxia can occur when an animal is removed from water, because the gills can collapse. Decapods are exposed to air during damp storage, but also sometimes whilst awaiting transfer to vehicles or storage containers. For example, a study of crabs transported from the UK to Portugal found that crabs held in the buckets for longer showed increased haemolymph L-lactate, acidity, and haemocyanine before the journey, and lasting lowered haemolymph pH throughout the entire journey (Barrento *et al.* 2010). Since hypoxia can also occur in viviers with seawater low in oxygen, in some cases, decapods will have equivalent or even better experiences in damp storage than storage in poor-quality seawater (Lorenzon *et al.* 2008; Barrento *et al.* 2011, 2012). However, the focus should be on ensuring higher-quality seawater transport, good water quality, and appropriate temperature. Keeping seawater clean and well-aerated can be challenging but is very important (Jacklin & Combes 2005; Seafish *et al.* 2024). However, where this is not possible, it may sometimes be preferable to use temporary damp storage. The maximum duration of damp storage should be investigated for key species to help prevent suffering.

#### Lack of food

Decapods in medium-term storage, such as lobsters, are often not fed (Seafish *et al.* 2024), partly to prevent contamination and soiling of the water with uneaten food and waste products. They can survive without obvious weight loss or increased mortality risk for several weeks without food (e.g. Siikavuopio *et al.* 2018), although there are species differences (Sacristán *et al.* 2017). In the wild, decapods have periods of fasting as part of their moult cycle (Lipcius & Herrnkind 1982). Recently moulted decapods are usually avoided in the fishing industry, because their flesh is very watery and their soft shells make them vulnerable to damage, so stored individuals will mostly comprise animals between moults that would be motivated to feed, and a smaller proportion that may have been preparing to moult and therefore would not feed. Housing animals in colder water may help them cope with lack of food, as indicated by physiological markers (Albalat *et al.* 2019; Siikavuopio *et al.* 2018). The resilience to starvation at cool temperatures in terms of bodyweight and mortality suggests that lack of food might pose little welfare concern for some species over the short- to medium-term, although this has not been tested directly, and fasting does confer some gradual physiological effects.

#### Lack of access to dark shelters

Decapods in the wild will spend substantial amounts of time in dark shelters. Given a choice between a light area and a dark shelter, crabs will typically prefer the dark shelter (e.g. Barr & Elwood 2011; Hamilton *et al.* 2016). Crayfish (*P. clarkii*) in an anxiety-like state will avoid bright areas (Fossat *et al.* 2014, 2015). Given this aversion to light, good practice for handling decapods must involve providing them with access to dark environments. This is already recommended by Seafish as one of their “10 golden rules” for handling crustaceans and in their codes of practice (Jacklin & Combes 2005; Seafish *et al*. 2024). Yet there is evidence (obtained by the campaign group Crustacean Compassion) that supermarkets selling live lobsters in the UK commonly do not provide access to dark shelters (Carder 2017) and display lobsters under bright lighting (Crustacean Compassion 2020).

#### Live purchase

Live decapods can be ordered from Amazon and other online retailers in countries such as the UK and US, sometimes travelling internationally. There is no way to ensure welfare-sensitive handling when a live animal is delivered to a private home. This practice carries an inherent risk of poor handling and inappropriate slaughter methods (see also the following section). Ending this practice would be a simple intervention to avoid this specific set of risks for poor decapod welfare.

A report by the campaign group Crustacean Compassion (2020) described highly inconsistent advice given to customers purchasing live lobsters in UK wholesalers on how to effectively transport, store or slaughter the animals. There is a need for enforceable codes of good practice regarding the advice and training that is provided in these settings. Live animals should only be sold to customers who are trained in appropriate handling and slaughter methods. Industry codes of practice suggest that animals should only be dispatched by competent persons (Seafish *et al.* 2024), but currently it is difficult to define or enforce this.

### Stunning and slaughter

One of the biggest threats to decapod welfare is inhumane methods of stunning (causing loss of consciousness – conditional of course on the presence of conscious experience) and slaughter (Conte *et al.* 2021). While electrical stunning seems to be the most promising method for causing loss of consciousness, this is still not in widespread use and the loss of consciousness (as opposed to immobility and unresponsiveness) has not been confirmed. The best mechanical slaughter methods (double-spiking and whole-body splitting) require highly trained operators and are not viable on a large commercial scale. Other methods, including live boiling, chilling, dismemberment, and osmotic shock, seem very likely to cause prolonged suffering before death. Asphyxiation, which is the most common method used for wild-caught shrimp and in many shrimp farms globally, has not been studied but is highly unlikely to be humane. The following sections discuss these methods in detail and identify the most pressing evidence gaps.

#### Stunning

Effective stunning not only immobilises the animal but also abolishes sentience (see discussion in Conte *et al.* 2021). Electrical stunning has the potential to be an effective method. Electric shocks of sufficiently high voltage and long duration can stun (and, at even higher voltages or longer durations, kill) crustaceans. Pre-dispatch stunning is a legal requirement in New Zealand and Switzerland. There are now several manufacturers producing electrical stunning equipment (e.g. Crustastun® [Mitchell and Cooper Ltd, Uckfield, UK] and Polar Systems Ltd [Kings Lynn, UK]) and include both single-animal units for the hospitality sector and a large-scale stunner for processors.

Electrical stunning appears to be the most humane method for stunning and/or slaughter. Roth and Øines (2010) compared electrical stunning, chilling, boiling, and CO_2_ gassing for slaughter of crabs (*C. pagurus*), and found that electrical stunning was the only method effective within 1 s. Crustastun® units are effective for a range of species, such as brown crabs (*C. pagarus*), lobster (*H. gammarus*), Norway lobster (*N. norvegicus*), and shore crab (*Carcinus maenas*), rapidly causing loss of consciousness (as measured by cessation of behavioural and neuronal activity) without creating stress (as measured by sampling L-lactate in haemolymph and occurrence of autotomy [Neil 2010, 2012; Neil & Thompson 2012; Neil *et al.* 2022]; though other work [Roth & Øines 2010; Roth & Grimsbø 2016] has found autotomy in response to electroshock of insufficient voltage). We note that only the first of these two latter studies has been peer-reviewed. In other work, electric shock immobilised and reduced heartrate in *P. clarkii* and *Litopenaeus vannamei* (Weineck *et al.* 2018) A non-peer-reviewed study also found that Crustastun® reliably kills Norway lobster (Albalat *et al.* 2008).

However, a degree of caution is needed in interpreting these results. While they suggest that Crustastun® did not cause extreme physiological stress, we cannot conclude from this that it is painless (Stevens *et al.* 2016). Stress responses can indicate pain (Elwood 2016), but this study should be considered in the context of how Crustastun® affects other (neural) indicators. A lack of significantly increased haemolymph lactate or autotomy, especially in experiments with small sample sizes, is insufficient evidence that high-voltage shocks do not induce pain. Elwood and Adams (2015) found that shore crabs (*C. maenas*) exposed to a weak electric shock for a short time exhibited higher levels of haemolymph lactate than controls. Fregin and Bickmeyer (2016) found that the Crustastun® induced a seizure-like pattern of increased neural activity, combined with an absence of responsiveness to mechanical stimulation (interpreted as total anaesthesia), but when crayfish were dropped into boiling water after stunning, the neural response – though much reduced relative to controls – was not abolished. We do not know what the seizure-like neural activity induced by electrical stunning feels like for decapods though, in humans, epileptic seizures involving the whole brain (as opposed to a specific region) result in loss of consciousness, so the same may be true for these animals.

Pharmacological anaesthesia is a potential alternative to electrical stunning, despite being limited to chemicals safe for human consumption. Two prime candidates are clove oil and AQUI-S®, a clove oil-based product without the former’s odour. In both, the active ingredient is eugenol (4-allyl-2- methoxyphenol). To our knowledge, pharmacological anaesthetics are rarely used on crustaceans in the UK. However, as a fish anaesthetic (Soto 1995; Anderson *et al.* 1997; Keene *et al.* 1998), AQUI-S® has been approved for human consumption in New Zealand, Australia, Chile, South Korea, Costa Rica, Honduras, and Norway, but not the EU or US (Priborsky & Velisek 2018).

Several studies suggest that clove oil and AQUI-S® stun crustaceans, though this can take some time. Eugenol temporarily immobilises blood-spotted crabs (*Portunus sanguinolentus*) in 14 min (Premarathna *et al.* 2016), and Australian giant crabs (*Pseudocarcinus gigas*) in 30 min (Gardner 1997) but up to 188 min in hairy shore crabs (*Hemigrapsus oregonensis*) (Morgan *et al.* 2001). Eugenol also immobilises other crustaceans, including lobsters (*H. americanus*: Waterstrat & Pinkham 2005), langoustine (*N. norvegicus*; Cowing *et al.* 2015), crayfish (*Cherax quadricarinatus*; Ghanawi *et al.* 2019), prawns (*Macrobrachium rosenbergii*; Coyle *et al.* 2005) and shrimps (*P. monodon*; Cai *et al.* 2012) (for more detailed reviews, see de Souza Valente [2022], Spoors *et al.* [2023] and Rotllant *et al.* [2023]). However, these pharmacological studies typically use behavioural indicators of stunning, which do not distinguish anaesthesia from paralysis. Eugenol’s mode of action is also poorly understood. Whilst pharmacological anaesthetics are potentially effective, more research is needed.

Chilling is another stunning technique, sometimes used for transport (as discussed previously in *Handling of wild-caught decapods during capture, transport and sale*). As crustaceans are ectothermic, they enter a state of torpor when external temperatures drop below a certain threshold. This renders them immobile, preventing autotomy and aggression between individuals. Torpor also facilitates nerve centre destruction, allowing a faster and more humane dispatch. Decapods are typically chilled by placing them into cold water or ice slurry. However, it is unclear whether chilling-induced inactivity is associated with loss of consciousness. Lobsters (*H. gammarus* and *H. americanus*) and crayfish (*Astacus astacus* and *A. leptodactilus*) both showed neural activity after an hour in cold water or ice slurry (Fregin & Bickmeyer 2016). Recordings of the number, amplitude, and rate of nerve impulses in *Nephrops* after 30 min of being immersed in ice were virtually the same as in the control animals, indicating no reduction of brain activity (Albalat *et al.* 2022a). Blue crabs (*C. sapidus*), red swamp crayfish (*P. clarkii*), and white-leg shrimp (*L. vannamei*) held in ice slurry showed decreased heart rate, although most crabs still had a heart rate after five minutes and exhibited central neural processing for muscle reflexes after two minutes (Weineck *et al.* 2018). The effectiveness of chilling, even for inducing immobility, depends a lot on the cold tolerance of the particular species and appears to be ineffective for some (Rotllant *et al.* 2023). As discussed previously, there is also the potential for negative welfare experience associated with chilling. More research is needed to establish whether chilling itself is painful, but the existing literature suggests that cold-induced immobilisation leaves crustaceans susceptible to pain from subsequent procedures. Use of slush-ice presents another welfare concern: salinity drops as the ice melts, which can lead to osmotic shock before torpor is induced, although maintaining salinity can resolve this issue (AHAW 2005).

From a welfare perspective, crustaceans should be stunned before dispatch, and this is recommended in UK industry codes of practice (Seafish *et al*. 2024). Electric and pharmacological stunning are the most promising approaches. Future research could identify ways to make stunning more practical and effective. The Humane Slaughter Association is currently funding research into effective methods of stunning and slaughtering crustaceans. Chilling is not a humane stunning method if it merely paralyses crustaceans without anaesthetising them. This method has already been banned in Switzerland and parts of Italy.

#### Mechanical slaughter (dispatch)

The shellfish industry uses the term ‘dispatch’ to refer to the slaughter of decapods; here, we use the two terms interchangeably. Unlike vertebrates, crustaceans have a decentralised nervous system. Crabs have two main nerve clusters (ganglia), and lobsters have 13 interconnected ganglia down the ventral nerve cord. The result is that methods that target only the brain will not necessarily kill the animal quickly (Roth & Øines 2010).

Spiking involves piercing the underside with a spike, destroying the ganglia. This method is recommended for crabs, because the brain (or cerebral ganglion) and ventral nerve mass (or thoracic ganglion) can both be spiked in rapid succession in a procedure known as ‘double spiking’. An early study for the Universities Federation for Animal Welfare (UFAW) recommended double spiking as the most humane method for slaughtering crabs (Baker 1955). Although double spiking is relatively quick, it is not instantaneous. At present, most UK crab processors only destroy one ganglion (‘single spiking’). Single spiking creates a welfare risk because it is less likely to kill the animal quickly and reliably (Roth & Øines 2010). UK codes of practice recommend double-spiking following stunning of crabs (Seafish *et al.* 2024), but regulations requiring double spiking (coupled with education about why this matters) would improve UK welfare standards; as would similar regulations globally.

Spiking is unsuitable for lobsters, because their chain of ganglia cannot be individually pierced quickly and accurately. To destroy all 13 ganglia, lobsters’ under-surface must be severed down the longitudinal midline using a knife. This process, known as splitting, is common in restaurants (industry sources) and is the recommended dispatch method following stunning according to UK industry guidance (Seafish *et al.* 2024). Due to the demand for whole lobsters, chefs typically only split the head (head splitting), rather than the whole body (complete splitting). However, head splitting leaves the posterior ganglia intact, raising the chance of continued survival. We cannot be confident that head splitting reliably abolishes consciousness immediately. From a welfare perspective, lobsters should be split from head to tail, destroying all 13 ganglia and killing the animal. Whole-body splitting should take less than 10 s when performed by a skilled practitioner.

Tailing involves separating the thorax from the abdomen. On *Nephrops* (langoustine) vessels, for instance, the abdomen is usually twisted away from the thorax (industry sources). Large vessels may chill the *Nephrops* beforehand, inducing immobility but without necessarily abolishing consciousness. As well as *Nephrops*, crayfish are slaughtered using tailing in the UK (industry sources). While spiking and splitting (properly performed) destroy all the animal’s ganglia, tailing does not. For this reason, it should not be recommended without prior effective stunning.

High-pressure processing involves crushing batches of crustaceans. It is claimed that high- pressure processing kills crustaceans in < 6 s, equivalent to spiking and splitting (industry sources). We have not been able to find robust scientific evidence confirming this. High- pressure processing without effective prior stunning has the potential to cause pain, even if it is over quickly. The use of this method varies by region – although it is the most common form of dispatch in the US, it is rare in the UK (industry sources).

Correctly practised, spiking and splitting are relatively quick dispatch methods. Quickly destroying every ganglion before further processing (e.g. boiling, freezing, or chopping up) ensures that the animal is dead and may not feel further pain. However, both tailing and routine spiking/splitting practices (especially single spiking and head splitting) do not destroy all ganglia. Double spiking crabs and completely splitting lobsters (as performed by competent and trained operators) should be considered best practice. Nevertheless, all mechanical dispatch methods take several seconds and may sometimes leave ganglia intact, especially when performed quickly or by untrained personnel. Crustaceans should therefore be effectively stunned beforehand.

#### Chilling

Decapods are sometimes dispatched using extremely low temperatures in refrigerators, freezers, or on ice. The welfare issues outlined in the section on stunning also apply here: nervous system activity continues after chilling, melting slush-ice can cause osmotic shock, and death is slow. Gardner (2004) argued that this method of dispatch is slow, inconsistent, and aversive. However, there is currently no evidence for cold-sensitive nociceptors in crustaceans (Puri & Faulkes 2015). If future research confirms their absence at more realistic temperatures in more species, low temperatures could conceivably represent a humane method of slaughter.

Chilling is a rare slaughter method, because it reduces meat quality (industry sources; but see Albalat *et al.* 2022a, who found no significant effect), but is common in domestic kitchens. This is concerning as, unlike commercial blast freezers, home freezers do not reduce temperature rapidly. Crustaceans in home freezers must, therefore, be left to die over a period of more than 1 h (Roth & Øines 2010). Edible crabs autotomise during freezing, indicating distress (Roth & Øines 2010). This prolonged suffering may be worse than rapid methods considered inhumane (e.g. boiling).

#### Boiling

Boiling is perhaps the most controversial dispatch method, having been banned in several jurisdictions (Switzerland, New Zealand, and parts of Italy). Immersion in boiling water is nonetheless common in UK restaurants and domestic kitchens for lobster, *Nephrops* (langoustine), small crabs, crayfish, shrimps, and prawns, as well as on-vessel for brown shrimp, although recent industry guidance has urged users to attempt to avoid processing or cooking crustaceans before stunning or killing them (Seafish *et al.* 2024).

Boiling elicits various behavioural and physiological symptoms of distress, such as unco-ordinated movements and escape attempts in crabs (*C. pagurus*; Baker 1955). More recent work on lobsters and cuttlefish did not observe such behaviours but did find that intense neural activity continued for up to 30–150 s after immersion (Fregin & Bickmeyer 2016). This suggests a period of up to 2.5 min (this duration aligns with an estimate by Roth & Øines [2010], obtained by a different method) of continued sentience, potentially involving extreme suffering. Smaller individuals died more quickly than larger ones, suggesting that boiling involves less prolonged suffering for smaller crustaceans (e.g. shrimps). This has recently been supported through work by Lauridsen and Alstrup (2024), who found more rapid heating curves for smaller species, suggesting they may reach stunning or killing temperatures in under 10 s; compared with several minutes for larger species.

To address welfare concerns regarding live boiling, a number of authors have recommended immersing crustaceans in cold water and slowly raising the temperature (e.g. 1°C per min). Evidence on the effectiveness and welfare effects of this method are mixed. Some studies have found that crabs, lobsters, and crayfish do not show behavioural responses indicating pain and distress (e.g. tail-flipping or escape behaviour; Gunter 1961; Fregin & Bickmeyer 2016) and that CNS activity disappeared above 32°C in lobsters (*H. gammarus* and *H. americanus*) and crayfish (*A. astacus* and *A. leptodactilus*) (Fregin & Bickmeyer 2016). However, other studies found that edible crabs (*C. pagurus*; Baker 1955) and red swamp crayfish (*P. clarkii*; Adams *et al.* 2019) displayed behaviours indicating distress, including escape attempts, unco-ordinated movements, and autotomy; and crayfish still showed a heartbeat and functional nervous system up to 44°C even when apparently unresponsive (Adams *et al.* 2019). Hence, a lack of behavioural responses to boiling may not indicate a loss of consciousness. This evidence is therefore insufficient to suggest that gradually raising water temperature (without prior stunning) is more humane than dropping an animal into boiling water. There is still a serious risk that it causes suffering over a period of minutes.

#### Freshwater immersion

Crustaceans immersed (‘drowned’) in freshwater must usually be left overnight. This practice is rare in the UK, as it reduces meat quality, but is sometimes practised on lobster and brown crab (industry sources). From a welfare perspective, it cannot be recommended. Crabs immersed in freshwater have shown behavioural signs of distress, such as unco-ordinated movement and increased respiration (Baker 1955), and even autotomising and tearing at their own legs and abdomen (Gardner 1997). Like chilling, freshwater immersion potentially leads to more prolonged suffering than faster methods considered inhumane, such as boiling.

### Aquaculture

Farming of decapods is increasing worldwide, partly due to challenges with the sustainability of fisheries, but also because the knowledge and technology required for successful decapod aquaculture has reached a point where it is a viable option for many species. In 2017, the number of individual decapods farmed for food was estimated at between 255 and 605 billion, with approximately 85% being shrimp and prawns (Mood & Brooke 2019). As of 2022, aquaculture contributed 68% of crustacean production, with 6.8 million tonnes of whiteleg shrimp representing the predominant species (FAO 2024). In most systems, decapods are bred and reared to marketable size within captivity; whereas in other systems, such as for some lobsters, decapods are hatched and reared as larvae, but then released and ongrown to replenish fisheries, where they may later be caught as adults. A minority of decapods or their ‘seed’ used in aquaculture are sourced from the wild. Aquaculture facilities can range from extensive open systems (connected with an external water body, such as the sea or rivers) to intensive closed systems (which may involve water recirculation through filters). The potential welfare implications of commercial practices in decapod aquaculture vary across species, systems and countries, and practices are evolving especially rapidly for some of the more newly cultured species. Many of the aforementioned environmental stressors that present welfare risks to live-caught decapods in wet storage, such as poor water quality and overcrowding, can also apply to farmed decapods, and over a longer timescale.

#### Shrimps

Most of the decapods used in aquaculture are shrimp, with almost 10 million tonnes produced per year under increasing intensification (Wuertz *et al.* 2023). The most commonly farmed species is the whiteleg shrimp (*L. vannamei*). While there is currently little evidence one way or the other regarding sentience in penaeid shrimp (Birch *et al.* 2021), a precautionary approach based on the lack of research and their close taxonomic relationship to other decapods for which there is more convincing evidence means their welfare should be taken seriously and potential harms prevented where possible. Alongside problems of humane slaughter discussed above, one of the most pressing problems in shrimp aquaculture is the practice of eyestalk ablation, which poses a severe welfare risk if the animals are sentient.

Eyestalk ablation is a controversial practice that involves removing one or both of the eyestalks of a mature broodstock female prawn in order to induce egg production. It has a range of negative effects on the animals (for a review, see Albalat *et al.* 2022b) and could be linked to suffering. Eyestalk ablation in *L. vannamei* causes recoil reactions (Taylor *et al.* 2004) and in *M. americanum* causes tail-flicking and rubbing of the wound site (Diarte-Plata *et al.* 2012), all of which are dampened by the use of anaesthetic (lidocaine). In recent years, experiments with ablation-free approaches by Zacarias *et al.* (2019, 2021) have suggested that eyestalk ablation may not be necessary for economically viable shrimp aquaculture, and that avoiding it leads to better reproductive performance from the breeding females and more resilient offspring with lower mortality rates. Banning eyestalk ablation will be a crucial part of high-welfare shrimp aquaculture. As it does not appear that shrimp aquaculture companies in the UK use eyestalk ablation (industry contacts) there would be no major downside to banning eyestalk ablation there, but any immediate welfare benefit would be limited. In the UK, the welfare benefits of such a ban would be limited due to the small size of the industry and the fact that UK shrimp aquaculture companies do not appear to use eyestalk ablation (industry contacts), though for the same reasons there would also be no major downside to a ban. However, in other regions where the practice is still common, such as the US and Asia, such bans could have a greater impact.

Alongside this direct welfare harm, shrimp can show physiological and behavioural signs of distress when housed under inappropriate conditions, such as inappropriate salinity, low oxygen, high water turbidity, low temperature, and high stocking density (which can even lead to cannibalism) (Albalat *et al.* 2022b; Pedrazzani *et al.* 2023; Wuertz *et al.* 2023). Diseases are also common and have clear welfare implications depending on the disease (Albalat *et al.* 2022b). Some diseases are non-transmissible and can be caused by suboptimal environmental conditions, toxicity or nutritional deficiencies. Others are caused by a variety of pathogens and parasites and can lead to epidemic outbreaks that lead to mass mortality. The most common diseases include white spot disease, yellow head virus, and *Vibrio* spp bacterial infections (El-Saadony *et al.* 2022).

To prevent welfare issues, as well as the obvious production losses, there are many alternative actions that may be taken, which have been reviewed elsewhere (Seethalakshmi *et al.* 2021; Abdel-Latif *et al.* 2022; El-Saadony *et al.* 2022). Prophylactic antimicrobials may be added to the ponds via the feed, but these can lead to resistant strains of pathogens that can ultimately harm the shrimps and other species, including humans. Probiotics, prebiotics, vaccines and other biotechnological solutions have been suggested as more sustainable alternatives for the future, and their impact on shrimp welfare should be taken into account during development (Seethalakshmi *et al.* 2021). Ultimately, because disease risk is increased by use of high stocking densities, excessively warm temperatures, and poor water quality, farming shrimp under optimal conditions for their health and welfare and using good biosecurity practices will help prevent diseases and their associated welfare compromises. This can be difficult in practice and work will be required to determine optimal conditions that are also feasible in reality.

Lethal ‘stress tests’ carried out on small samples of larvae to check the quality of the larvae batch carry obvious welfare harms if larvae are sentient, through exposure to environmental stressors such as changes in salinity and temperature or exposure to toxins such as ammonia and formalin (Wuertz *et al.* 2023). Harvesting of animals involves stressful capture using nets or pumps, which triggers flight behaviour and physiological stress responses (Wuertz *et al.* 2023).

Historically, shrimp standards and best practice guidelines have incidentally included welfare components, such as disease, stocking density, and water quality, but this has not been their focus (e.g. Aquaculture Stewardship Council [ASC] 2023). Since publication of our original report, however, several welfare-based best practice guidelines and reviews have been published (Albalat *et al.* 2022b; Crustacean Compassion 2023; Pedrazzani *et al.* 2023; Shrimp Welfare Project [SWP] 2024). Moving forward, we recommend further strengthening these guidelines, using the peer-reviewed literature to make more specific recommendations, and (where this is lacking) carrying out welfare science to build a better evidence base. This would complement research into the development of shrimp sentience across all life stages.

#### Lobsters, crayfish and crabs

Crab, crayfish and lobster farming occur on a smaller scale than shrimp farming, but are increasing rapidly. Of global crustacean aquaculture production in 2022, the second most common species after the white leg shrimp at 62.2%, was the red swamp crayfish (*P. clarkii*) at 23.3%, followed by mitten crabs (*Eriocheir* spp) at 6.4% (FAO 2024). Many of the welfare risks associated with aquaculture of crabs, crayfish and lobsters are shared with those of shrimps. Disease is again a significant threat to the welfare of these animals, with the specific diseases differing somewhat between species, and being most common in intensive systems with suboptimal environments and high stocking densities. Common diseases and parasites of farmed crabs, including the commercially important Chinese mitten crab (*E. sinensis*), mud crabs (*Scylla* spp), swimming crabs (*Portunus* spp), blue crabs (*C. sapidus*) and shore crabs (*C. maenas*), have been extensively reviewed by Coates and Rowley (2022). Depending on the specific disease, signs of poor welfare included lethargy, limb loss, tremors, loss of appetite, failure to moult and mortality. As with shrimp aquaculture, reducing stocking density and providing near optimal conditions are vital for preventing or minimising the spread of such diseases. Antimicrobial stewardship will be important here, as with shrimp aquaculture. One approach is to dip the tail or the whole of wild-caught berried female *H. gammarus* into antimicrobial solution before introducing them into a hatchery, but there are concerns that this may disrupt their microbiome (Hinchcliffe *et al.* 2022). Research is needed to further understand how to prevent and treat diseases in these, in some cases relatively newly cultured, decapod species.

There are also welfare risks associated with the demand for ‘soft-shell’ crabs. These are newly moulted crabs of species most commonly comprising mud crabs (*Scylla* spp) in Asia, the Atlantic Blue Crab (*C. sapidus*) in the US and the blue swimming crab (*Portunus pelagicus*) in India. For most of these species, there is only a short window of a few hours following moulting during which the crabs can be harvested for the soft-shell market. Therefore, there is commercial pressure to be able to induce moulting in these crabs, and although no single method is yet to prove entirely effective, many potential methods are being used and optimised, as reviewed by Waiho *et al.* (2021). Eyestalk ablation is one such method, which seems relatively ineffective and carries similar welfare risk to that in shrimps, so should be discontinued. Induction of moulting by removal of the walking legs, the claws, or both was compared in *Scylla olivacea* (Rahman *et al.* 2020); in that study, the claws were manually snapped off, whereas the legs were cut to induce autotomy. Ablation of both claws significantly hastened moulting and also appeared to increase crab body size following the moults. Crabs with limbs removed did not show significantly greater mortality rates than controls. However, no measures of animal welfare were included in that study, and as described previously in *Handling of wild-caught decapods during capture, transport and sale*, declawing is of animal welfare concern and has been shown to increase crab mortality in other studies (Waiho *et al.* 2021). Most other methods involve the injection of moult regulation hormones (ecdysteroids, e.g. E20), phytoecdysteroids, melatonin or other substances; these can be effective, although they are still at the experimental stage and the effects on crab welfare unknown (Waiho *et al.* 2021). Feeding crabs sufficiently also hastens moulting compared with restricted food rations (Gong *et al.* 2022). A further cause for welfare concern for soft-shell crabs is that, when harvested, they are usually placed into –20°C while still alive to be frozen before their shells harden. The welfare risks of freezing as a slaughter method, especially without prior stunning, are described above (Waiho *et al.* 2021).

The welfare needs of species that are relatively new to aquaculture may not yet be well understood, creating welfare risks as industry develops. For example, insufficient or inadequate food may impair growth, risk deficiency diseases and hunger, and potentially cause cannibalism (Harlıoğlu & Farhadi 2017; Hinchcliffe *et al.* 2022; Nankervis & Jones 2022). It was noted that individually housed *H. gammarus* larvae grew more slowly than communally housed ones fed the same ration of dry pellets, which are not readily accepted by the animals; this implies that communally housed larvae may have supplemented their diet – presumably through cannibalism. Unlike many shrimp species, some decapod species also require darkness or shelters to thrive and reduce aggression (Shelley & Lovatelli 2011; Yu *et al.* 2020; Zhang *et al.* 2022). It will be important that research and development activities in decapod aquaculture evaluate welfare-relevant metrics beyond solely production or economic outcomes. This requires careful selection of species with fewer potential welfare risks (Chiang & Franks 2024) as well as development of species-specific welfare guidelines prior to commercial-scale use (for a recent example of the development of a comprehensive welfare index for whiteleg shrimp [*P. vannamei*], see Pedrazzani *et al.* 2024).

Where they are farmed, the concerns for handling and transport described in the previous section will apply. There are also some additional concerns regarding appropriate housing conditions. For instance, insufficient holding space for rearing lobsters can slow growth, and limit development of a normal behavioural repertoire (Latini *et al.* 2023).

## Recommendations and evidence gaps

The identified welfare issues for cephalopods and decapods lead to several clear recommendations for immediate, cost-effective interventions.

Banning declawing and nicking in crabs would be a positive step, given credible evidence that they cause suffering. Declawing was banned in the UK from 1986 to 2000, and banning this practice both from fisheries and in aquaculture would be an effective intervention to improve crab welfare. Developing and implementing practical alternatives to nicking should also be a priority for research. Moreover, addressing the sale of decapods to untrained handlers (especially through online retailers, where there is no face-to-face contact between supplier and purchaser) is vital for preventing welfare risks associated with improper handling and inhumane slaughter methods.

High-welfare octopus farming appears to be extremely difficult, if not impossible, to implement. Despite the absence of commercial-scale farms at present, impending projects like those proposed by Nueva Pescanova in Spain indicate the emerging reality of this industry. Proactive measures like implementing bans on octopus farming in countries where it is likely to proceed (e.g. Spain, Australia, Japan, Mexico), or restricting imports in regions less inclined to farm, would be proportionate ways to err on the side of caution (Birch 2024).

Aquaculture of decapods is clearly established, especially for shrimps, but many welfare risks exist and improved practice is needed. For many farmed decapod species, basic information on optimal environmental conditions, feeding, and stocking densities to prevent disease and poor welfare is still required. Practices such as eye-stalk ablation and limb removal pose high risks to welfare and appear largely ineffective, so should be banned. The welfare impacts of anti-microbial and hormone applications require assessment compared with alternatives. The current lack of clear and enforceable regulations for the handling, housing, and slaughter of decapods often leads to outdated or cruel practices. Establishment of such standards is urgent.

There are several critical gaps in our current understanding that urgently require research to improve best practices in the handling and processing of cephalopods and decapods. However, it is crucial to keep in mind the same welfare considerations when performing research. Much of the research into animal sentience and welfare can inflict many of the same harms discussed here (Baker *et al.* 2024), associated with capture, housing, (lack of) anaesthesia, and inhumane slaughter methods. While it may occasionally be necessary to inflict harms during research to gain information that will lead to greater long-term benefits, researchers should take every precaution to prevent or minimise these harms.

A key area is the development of humane stunning and slaughter methods for these animal groups. For cephalopods, the only currently approved method for humane slaughter is an anaesthetic overdose, which is unsuitable for animals intended for human consumption. Mechanical methods like brain cutting or puncturing are not only time-consuming and skill-intensive but also questionably humane. Research into immediate post-catch humane slaughter methods for cephalopods, which align with commercial usage, is therefore essential.

For decapods, methods such as double-spiking (for crabs), whole-body splitting (for lobsters), and electrocution with specialised devices are considered potentially humane. However, these methods take 10–15 s to execute and require specific skills. Prioritising research to develop reliable and humane killing methods for decapods within a shorter time-frame is critical, as recognised by the Humane Slaughter Association. Further research into effective methods of rapid stunning and humane slaughter for various commercially important decapod species and particularly smaller species like shrimps, is crucial.

Another significant research area is analgesia for both cephalopods and decapods. The effectiveness of drugs, including morphine, in managing or preventing pain in decapods remains unconfirmed (Rotllant *et al.* 2023). Research into anaesthesia is also limited, although some promising local and general anaesthetics have been identified (Butler-Struben *et al.* 2018; de Souza Valente 2022; Rotllant *et al.* 2023). Distinguishing between chemicals that immobilise animals and those that genuinely induce loss of consciousness is crucial, benefiting not just commercial species but also those used in research.

Additionally, it is vital to understand the diverse welfare needs of decapods, such as the optimal temperature ranges, diets, and stocking densities for housing different species. The new Seafish welfare codes of practice (Seafish *et al*. 2024) are an upgrade on previous guidelines (Jacklin & Combes 2005; see also Boyd *et al.* 2002; Shelley *et al*. 2011) that focused on more product quality than welfare. However, they still provide only general guidance for handling a range of species and we believe they still do not go far enough to address all the necessary considerations (e.g. humane slaughter). More welfare-centric, species-specific guidelines are still needed.

Practices such as storing decapods on ice raise questions about their thresholds for entering torpor, whether this state affects the state of consciousness, and if direct contact with ice activates nociceptors for cold temperatures. Establishing evidence-based maximum stocking densities and bulk weights for transportation can prevent issues like crushing and hypoxia. For cephalopods, developing best-practice guidelines for capture, transport, breeding, housing, and husbandry outside scientific contexts also need to be developed (for scientific contexts, see Fiorito *et al.* 2015). The development and implementation of such guidelines is important for ensuring the welfare of cephalopods in commercial settings.

Finally, there is an urgent need to develop and validate welfare indicators for all these species. Current indicators often focus on health, production status, or physiological stress (Paterson & Spanoghe 1997; Albalat *et al.* 2022b; Conneely & Coates 2023; Wuertz *et al.* 2023) rather than the animals’ affective experiences. Although recent research aims to assess the welfare of cephalopods and decapods (see e.g. Narshi *et al.* 2022; Andrade *et al.* 2023; Pedrazzani *et al.* 2023), a more extensive programme is necessary to effectively measure and improve welfare standards.

## Animal welfare implications and conclusion

The acknowledgment of cephalopod molluscs and decapod crustaceans as sentient beings has heightened concern for their welfare, particularly in light of certain commercial practices that pose potential welfare risks. This paper aims to make the animal welfare implications of these practices clear. By identifying practices with the highest welfare risks, we can initiate discussions on mitigating or preventing these risks. The recommendations proposed herein offer what we see as potentially practical, cost-effective strategies for immediate improvements in the welfare of cephalopods and decapods. We acknowledge the complexities attendant with any large-scale social or behaviour change, requiring education and engagement beyond mere legislative change. These suggestions should not be seen as a comprehensive account of how to mitigate or prevent all welfare risks to cephalopods and decapods, but rather initial suggestions of the more straightforward improvements. Furthermore, highlighting the most pressing evidence gaps should direct future research towards addressing the urgent welfare challenges facing these animals.

Changes to policy and regulation should not be seen as inherently opposed to the interests of those involved in the commercial use of cephalopods and decapods. As is already recognised for vertebrates, well-designed welfare regulations can *protect* producers from the erosion of standards that may occur when cost-cutting and welfare-reducing practices become commonplace in a competitive marketplace. These regulations can also reassure consumers who may be reluctant to purchase products associated with harmful or inhumane practices. Enhancing the welfare of cephalopods and decapods can be beneficial for the animals, producers, and consumers alike.
